# Increase in methicillin-resistant *Staphylococcus* spp. colonization among pregnant individuals during COVID-19 pandemic

**DOI:** 10.1038/s41598-024-64422-9

**Published:** 2024-06-28

**Authors:** A. Rio-Tinto, N. S. Costa, D. C. S. S. Alvim, L. M. A. Oliveira, T. L. R. De Oliveira, K. R. N. Dos Santos, S. E. L. Fracalanzza, L. M. Teixeira, P. Marinho, S. Taylor, S. Thomas, T. C. A. Pinto

**Affiliations:** 1 Instituto de Microbiologia Paulo De Góes, Universidade Federal Do Rio de Janeiro, Rio de Janeiro-RJ, Brazil; 2 Maternidade Escola da Universidade Federal Do Rio Janeiro, Rio De Janeiro-RJ, Brazil; 3 UK Health Security Agency, London, UK

**Keywords:** Antimicrobial resistance, COVID-19, Methicillin-resistant staphylococci, Pregnancy, SCC*mec* typing, Bacteriology, Applied microbiology

## Abstract

Methicillin-resistant *Staphylococcus* (MRS) has been associated with neonatal infections, with colonization of the anovaginal tract being the main source of vertical transmission. The COVID-19 pandemic has altered the frequency of antibiotic usage, potentially contributing to changes in the dynamics of bacterial agents colonizing humans. Here we determined MRS colonization rates among pregnant individuals attending a single maternity in Rio de Janeiro, Brazil before (January 2019–March 2020) and during (May 2020–March 2021) the COVID-19 pandemic. Anovaginal samples (n = 806 [521 samples before and 285 during the pandemic]) were streaked onto chromogenic media. Colonies were identified by MALDI-TOF MS. Detection of *mecA* gene and SCC*mec* typing were assessed by PCR and antimicrobial susceptibility testing was done according to CLSI guidelines. After the onset of the pandemic, MRS colonization rates increased significantly (*p* < 0.05) from 8.6% (45) to 54.7% (156). Overall, 215 (26.6%) MRS isolates were detected, of which *S. haemolyticus* was the most prevalent species (MRSH, 84.2%; 181 isolates). SCC*mec* type V was the most frequent among MRS (63.3%; 136), and 31.6% (68) of MRS strains had a non-typeable SCC*mec*, due to new combinations of *ccr* and *mecA* complexes. Among MRS strains, 41.9% (90) were resistant to at least 3 different classes of antimicrobial agents, and 60% (54) of them were *S. haemolyticus* harboring SCC*mec* V. MRS colonization rates and the emergence of multidrug-resistant variants detected in this study indicate the need for continuing surveillance of this important pathogen within maternal and child populations.

## Introduction

Staphylococci are abundant in the human microbiota, and the main sites of colonization are skin and mucous membranes^1^. *Staphylococcus aureus* is the most clinically relevant coagulase-positive species and one of the leading causes of community-acquired and nosocomial infections^2^. Coagulase-negative staphylococci (CoNS) cover a broader group of species, which can act as important opportunistic pathogens, mainly associated with immunocompromised patients, long periods of hospitalization, and medical procedures using indwelling devices^1^. In this scenario, CoNS stand out as a common cause of neonatal infections, mainly associated with the occurrence of neonatal sepsis, which is a frequent type of infection in newborns^3,4^. Among CoNS, *S. haemolyticus* has gained special attention, becoming the second most common CoNS isolated from nosocomial infections^5^, and predominantly found in neonatal intensive care units^3,4,6^.

Methicillin-resistant *Staphylococcus* (MRS) are of major importance in clinical settings due to its high rates of antibiotic resistance^1,2^. Methicillin-resistance gene *mecA* is located on a mobile genetic element called *staphylococcal* cassette chromosome *mec* (SCC*mec*) and encodes a penicillin-binding protein with low affinity to β-lactams, called PBP-2a (or PBP-2’), which results in broad-spectrum resistance to this class of antimicrobial agents^2^.

Anovaginal carriage of MRS strains during pregnancy can be a risk factor for development of early and late-onset disease in newborns, due to the possibility of vertical transmission^7^. As previously reported, several multidrug-resistant (MDR) bacteria can be transmitted from mother to newborn upon delivery, with MRS being a frequent cause of bloodstream infections in infants, particularly among those with low and very low birth weights^3,8^. During the COVID-19 pandemic, many studies have reported an increase in occurrence of MDR pathogens in human infections, which could have been, at least in part, fostered by the misusage of antimicrobial agents in this period^9,10^. Among the microorganisms that have been highlighted in this scenario, methicillin-resistant *S. aureus* (MRSA) and CoNS are included^10^.

Since 2008 our group assist the Teaching Maternity of Universidade Federal do Rio de Janeiro (ME-UFRJ) with the screening of pregnant individuals during routine antenatal care aiming at the detection of *Streptococcus agalactiae* (Group B *Streptococcus*, GBS) anovaginal colonization^11^. During the last years, we observed a gradual decrease in GBS colonization rates among this population, with a significant decrease after the onset of the COVID-19 pandemic^12^, suggesting the occurrence of ongoing changes in the anovaginal microbiota of the population analyzed. Considering the importance of staphylococci in neonatal infections, which can infect the newborn through vertical transmission, we expanded the anovaginal screening during pregnancy to also include isolation of *Staphylococcus* species with methicillin-resistance phenotype since 2019. Here we determined the anovaginal colonization rate and characteristics of MRS strains among pregnant individuals attending this same maternity in Rio de Janeiro, Brazil, considering scenarios before and during the COVID-19 pandemic, and observed a significant increase in colonization rates after the onset of COVID-19, with MDR *S. haemolyticus* carrying SCC*mec* V playing a prominent role in this scenario.

## Results

Between January 2019 and March 2021, surveillance conducted by swabbing was performed on all pregnant individuals (806) who attended the teaching maternity during antenatal routine care in that period. One clinical specimen was collected from each patient. Before the COVID-19 pandemic, 521 samples were collected, and during the pandemic period, 285. A total of 201 out of 806 (24.9%) patients were colonized by MRS. Of those, 14 (7%) presented colonization with two MRS species, and the rest presented colonization with only one species. Overall, 215 MRS strains were obtained, and nine species were identified, being *S. haemolyticus* the most prevalent (MRSH, 181; 84.2%), followed by *S. epidermidis* (MRSE, 11; 5.1%), *S. saprophyticus* (MRSS, 7; 3.3%), *S. sciuri* (MRSSc, 5;2.3%) and *S. aureus* (MRSA, 5; 2.3%).

**Figure 1:**
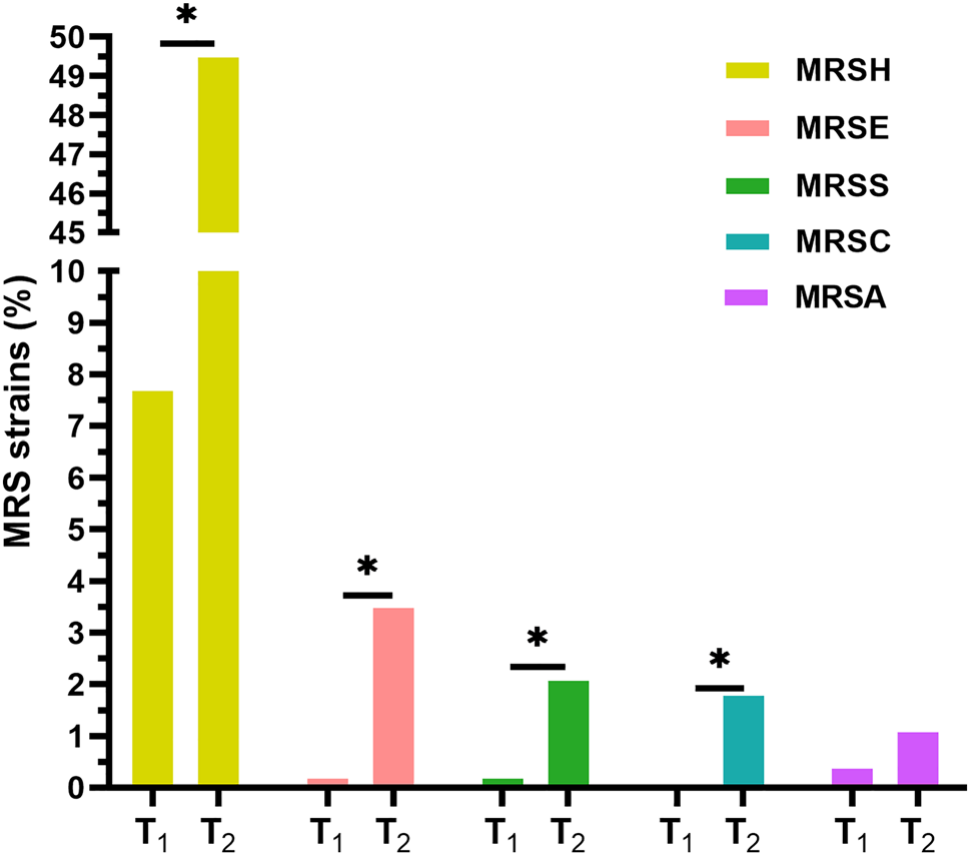
MRS species colonization rates before the onset and during the COVID-19 pandemic. MRSH = Methicillin-resistant *Staphylococcus haemolyticus*, MRSE = Methicillin-resistant *Staphylococcus epidermidis*, MRSS = Methicillin-resistant *Staphylococcus saprophyticus*, MRSSc = Methicillin-resistant *Staphylococcus sciuri,* MRSA = Methicillin-resistant *Staphylococcus aureus*. T_1_ = Before COVID-19 pandemic, T_2_ = During COVID-19 pandemic. *p value < 0.05.

MRS colonization significantly increased (*p* < 0.05) from 8.7% (45) to 54.7% (156) after the beginning of the COVID-19 pandemic. Colonization rates increased significantly (*p* < 0.05) for MRSH (7.7–49.5%), MRSE (0.2–3.5%), MRSS (0.2–2.1%) and MRSSc (0–1.8%). Although there was an increasing trend, a significant increase for MRSA colonization was not observed (*p* > 0.05) (Fig. 1).

**Figure 2:**
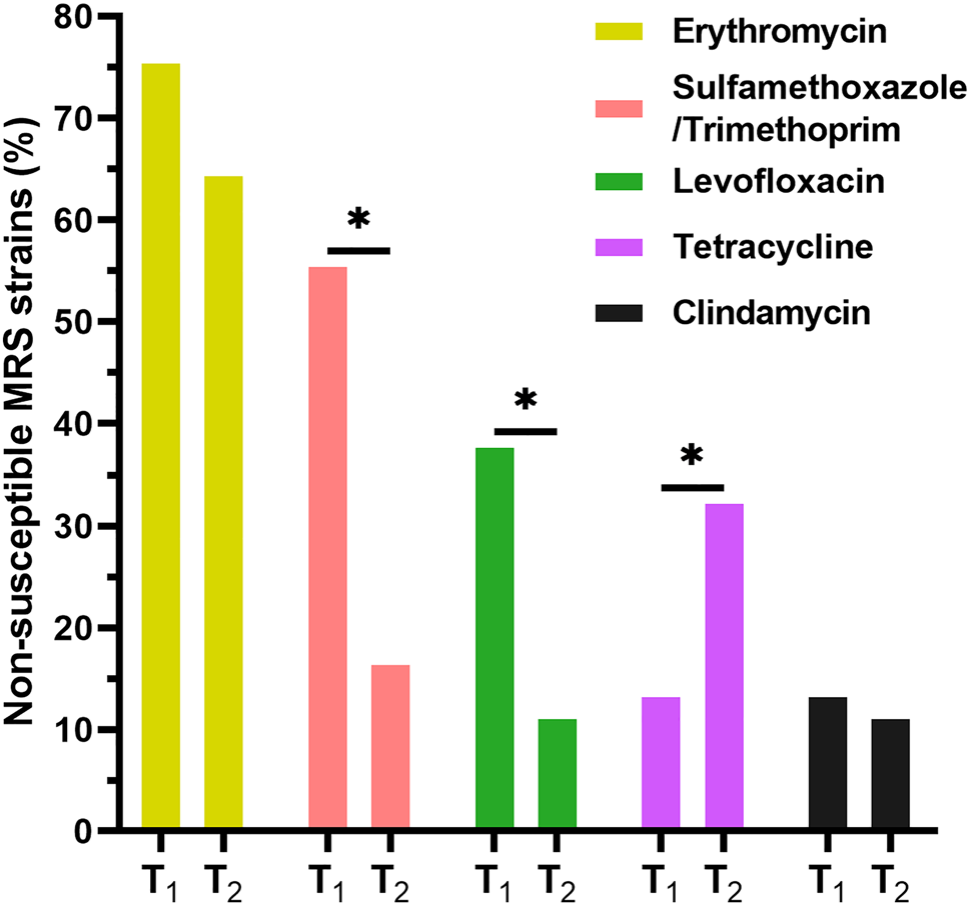
Non susceptibility rates for MRS strains before and during COVID-19 pandemic. T_1_ = Before COVID19-pandemic, T_2_ = During COVID-19 pandemic. *p value < 0.05.

Antimicrobial susceptibility testing showed a higher proportion of strains non-susceptible to erythromycin (70.7%; 152), followed by non-susceptibility to tetracycline (28.4%; 61), sulfamethoxazole/trimethoprim (24.6%; 53), levofloxacin (16.7%; 36) and clindamycin (11.6%; 25). After the beginning of the COVID-19 pandemic, non-susceptibility to sulfamethoxazole/trimethoprim and levofloxacin decreased significantly (*p* < 0.05) from 55.5% (25) to 16.5% (28) and 37.8% (17) to 11.2% (19), respectively, whereas non-susceptibility to tetracycline had a significant increase (*p* < 0.05) from 13.3% (6) to 32.3% (55) (Fig. 2). Non-susceptibility rates to erythromycin and clindamycin also decreased, from 75.5% (118) to 69.4% (34) and from 13.3% (19) to 11.2% (6), respectively; although not statistically relevant (*p* > 0.05) (Fig. 2). The distribution of antimicrobial susceptibility rates by species is presented in Table 1. Moreover, 19.5% (42) of strains showed iMLS_B_ resistance phenotype and 41.9% (90) were MDR. The iMLSB phenotype significantly increased (*p* < 0.05) from 4.4 to 23.5% during COVID-19 pandemic. However, the prevalence of MDR phenotype decreased significantly (*p* < 0.05) during the same period, from 57.8 to 37.6%.

**Table 1:** Distribution of antimicrobial susceptibility rates and SCC*mec* types among the five most prevalent species found in this study.

		All	Species				
			*S. haemolyticus*	*S. epidermidis*	*S. saprophyticus*	*S. aureus*	*S. sciuri*
Antimicrobial drug	Erythromycin	70.7% (152/215)	73.5% (133/181)	63.6% (7/11)	85.7% (6/7)	20% (1/5)	60% (3/5)
	Tetracycline	28.4% (61/215)	32% (58/181)	18.1% (2/11)	14.3% (1/7)	0% (0/5)	0% (0/5)
	Sulfamethoxazole/Trimethoprim	24.6% (53/215)	27.6% (50/181)	18.1% (2/11)	14.3% (1/7)	0% (0/5)	0% (0/5)
	Levofloxacin	16.7% (36/215)	19.3% (35/181)	9% (1/11)	0% (0/7)	0% (0/5)	0% (0/5)
	Clindamycin	11.6% (25/215)	9.4% (17/181)	9% (1/11)	0% (0/7)	0% (0/5)	100% (5/5)
MDR isolates		41.9% (90/215)	47% (85/181)	27.3% (3/11)	28.6% (2/7)	0% (0/5)	0% (0/5)
SCC*mec* type	II	2.3% (5/215)	1.1% (2/181)	0% (0/11)	14.3% (1/7)	0% (0/5)	20% (1/5)
	III	1.4% (3/215)	0% (0/181)	0% (0/11)	0% (0/7)	0% (0/5)	60% (3/5)
	IV	1.4% (3/215)	0% (0/181)	0% (0/11)	0% (0/7)	60% (3/5)	0% (0/5)
	V	63.3% (136/215)	70.2% (127/181)	45.5% (5/11)	28.6% (2/7)	0% (0/5)	20% (1/5)
	NT	31.6% (68/215)	28.7% (52/181)	54.5% (6/11)	57.1% (4/7)	40% (2/5)	0% (0/5)

MDR: multidrug resistance, NT: non-typeable.

**Figure 3:**
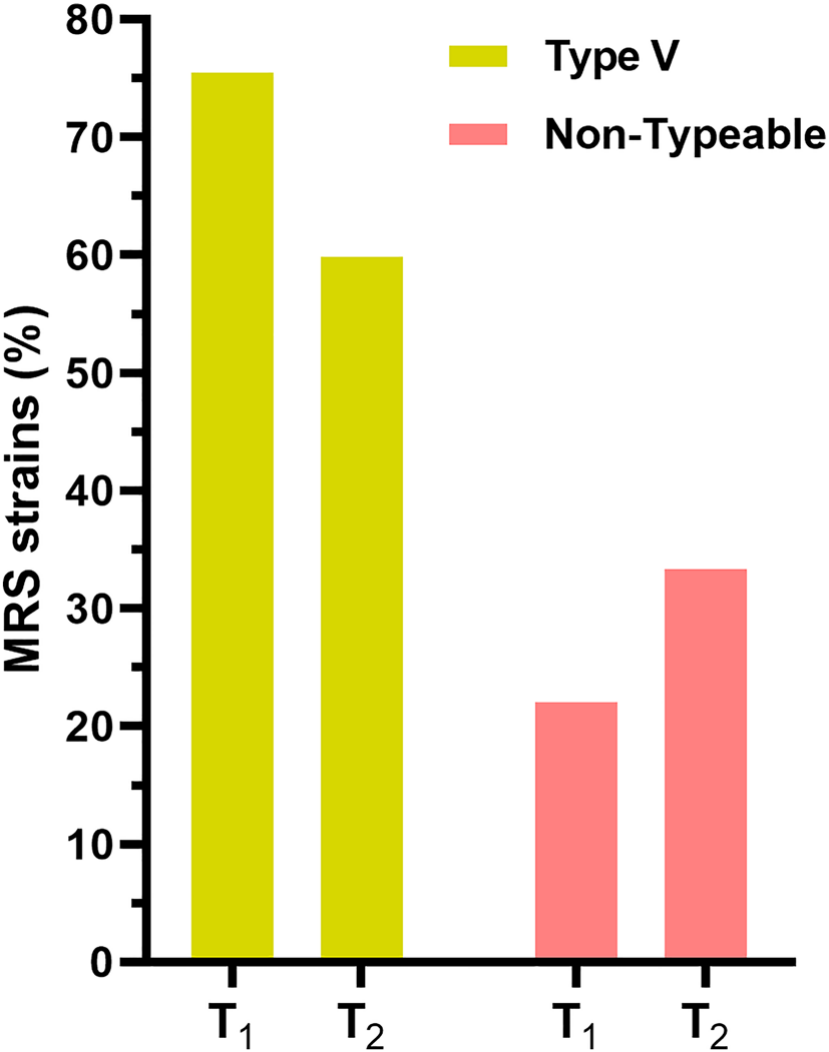
SCC*mec* type V and non-typeable distribution among MRS strains. T_1_ = Before COVID19-pandemic, T_2_ = During COVID-19 pandemic.

All 215 MRS strains recovered from chromogenic media were confirmed to be resistant to methicillin by cefoxitin/oxacilin disk-diffusion and harbored the *mecA* gene. SCC*mec* type V was the most prevalent (63.3%; 136), followed by type II (2.3%; 5), type III and IV (1.4%; 3). A large proportion of strains (31.6%; 68) could not be typed due to new combinations of *ccr* and *mecA* genes. Among those, 7 distinct combinations were identified, being the combinations *ccr* type 2/5 and *mecA* class C (18; 26.9%), *ccr* type 1/5 and *mecA* class C (10; 14.9%) and *ccr* type 2 and *mecA* class C (6; 9%) the most common. All other combinations presented less than 3 representative strains. The proportion of SCC*mec* non-typeable (NT) strains increased from 22.2% (10) to 34.1% (58) comparing before and during the pandemic, and the proportion of SCC*mec* type V strains decreased from 75.6% (34) to 60% (102) (Fig. 3). The distribution of SCC*mec* types by species is presented in Table 1.

## Discussion

*Staphylococcus* spp., highlighting the methicillin-resistant variants, are a main cause of nosocomial and community-acquired bacterial infections worldwide^5,13,14^, being emergingly associated with neonatal infections^15,16^. However, in resource limited countries, such as Brazil, where clinical and epidemiological surveillance practices during perinatal care are still scarce, these pathogens remain neglected. To our knowledge, this is the first study that evaluates the prevalence of MRS strains in anovaginal specimens during pregnancy in Brazil.

The most frequent MR-CoNS species in this study was *S. haemolyticus*, with a colonization rate of 84.2%. This species has a well-established role in harboring and transferring genetic material to other staphylococci, both CoNS and coagulase-positive staphylococci (CoPS)^17,18^. Hence, the presence of MRSH strains in the human microbiota can promote the dispersion of antimicrobial resistance determinants to other microorganisms colonizing the same niche. For MRSA, the anovaginal colonization rate (2.3%) found in this study was similar to the rate reported in other studies^19,20^.

Empiric treatment for COVID-19 patients was a common practice in many hospital settings worldwide, which included different antimicrobial agents such as macrolides and carbapenems^21,22^. In many countries, azithromycin was the most prescribed drug during this period^23^, including Brazil, where increased consumption of azithromycin was reported during the pandemic^24^. The abuse of antimicrobial agents is a well-known risk factor for the emergence of MDR pathogens^10,21,22^. Therefore, the significant increase in MRS colonization during the COVID-19 pandemic could be reflecting the impact of antibiotic overuse during this period.

Similar to our findings, other studies also reported high rates of non-susceptibility to macrolides in MRS, above 70%^25,26^, and non-susceptibility to tetracycline being considerably lower, between 19 and 26%^27,28^. For lincosamides, quinolones and sulfamethoxazole/trimethoprim, the non-susceptibility rates were below 25%, which are lower than other rates reported in the literature, all higher than 40%^13,25^. However, considering the overall antimicrobial susceptibility pattern, a great proportion of the tested strains were MDR. In this context, once again *S. haemolyticus* stands out, having the highest proportion (47%) of MDR strains among all MR-CoNS species identified in this study. This data highlights *S. haemolyticus* as an important CoNS associated with MDR phenotype, which has been pointed out in the literature in the last decades^5,13,27,29^, with reported MDR prevalence as high as 75.3% among this species^26^.

In this study, iMLS_B_ was a common phenotype among the tested strains, particularly those recovered during the pandemic. Since these strains may not respond well to clindamycin, potentially leading to treatment failure^30^, this finding emphasizes the importance of implementing the D-zone test in routine disk diffusion assays, especially in the clinical context, to ensure the detection of both constitutive and inducible resistance to clindamycin.

SCC*mec* typing is a common and well characterized typing method used in *S. aureus* strains. Most MRSA strains (60%) in this study harbored SCC*mec* IV, which has been reported as the most prevalent among MRSA strains associated with community-acquired infections in Brazil^14,31^. However, considering the possibility of a large variability of SCC*mec* elements in CoNS, applying SCC*mec* typing methods for this group becomes challenging, mostly because such methods were established for *S. aureus*. MRS-CoNS can either present new combinations of *mec* and *ccr* complex genes or present more than one *mec* and *ccr* complex in a single strain^29,32–34^.

In the present study, we detected a considerable amount of NT strains in MRS strains (31.6%), being as high as of previous reports, which ranged from 13.2 to > 50%^33,35,36^, with several strains having more than one *ccr* gene complex with a single *mec* gene complex. These findings were also reported by other groups,^29,32–34,37^ with the same combinations of *ccr* and *mec* gene complexes also found in our study, such as a *ccr* type 2 and *ccr* type 5 with *mecA* class C, and *ccr* type 1 and *ccr* type 5 with *mecA* class A.

Of note, 70.2% (127) of MRSH strains carried SCC*mec* type V. This association between MRSH and SCC*mec* type V in community-acquired MRS (CA-MRS) has also been reported by other groups, with MRSH type V rates varying from 14.2 to 55.5%^27,36–38^. Also, MDR phenotype was observed in several MRS strains (41.9%), of which 60% (54) were *S. haemolyticus* carrying SCC*mec* V.

As limitations of this study, we recognize that collecting anovaginal specimens from pregnant individuals at maternities from different locations could contribute to better comprehend and establish the epidemiological scenario and characterization of MRS colonization in this population, in Rio de Janeiro city. Moreover, the lack of data regarding methicillin-susceptible *Staphylococcus* colonization in our study sample may have impacted the comparison and analysis of MRS colonization dynamics among the whole *Staphylococcus* population colonizing the anovaginal site.

The increase in MRS colonization rate during the pandemic and the large number of strains with MDR phenotype observed in this study contribute to highlight the potential impact of the COVID-19 pandemic in the dynamics of bacterial infections, especially those associated with antimicrobial resistance. Thus, our findings point out to the need for developing and improving surveillance and public health policies for this bacterial group, mainly in special populations such as pregnant individuals and neonates, aiming to contribute to maternal and child health.

## Methods

### Clinical samples

This is a descriptive study on anovaginal colonization by methicillin-resistant *Staphylococcus* spp. during pregnancy in Rio de Janeiro, Brazil. Anovaginal specimens were collected from all pregnant individuals attending the ME-UFRJ, between January 2019 and March 2021, being separated in two scenarios according to the date of collection: before COVID-19 was declared a pandemic by the World Health Organization (January 2019–March 2020) and during the COVID-19 pandemic (May 2020–March 2021). Although clinical samples were collected constantly during the period evaluated, no samples were collected on April 2020 due to the COVID-19 lockdown. The ME-UFRJ is a specialized teaching maternity, providing outpatient care to individuals with high-risk pregnancies. The maternity is located in a major metropolitan area of Rio de Janeiro State, the city of Rio de Janeiro, in the Southeastern region of Brazil.

The specimens were obtained between the 35th and 37th gestational week during routine antenatal care, using the combined swab method according to previous guidelines^39^. All participants signed a consent form upon the sample collection. This project was approved by the Research Ethics Committee of UFRJ (Number: 43389321.9.0000.5257). The clinical samples were placed in cryogenic tubes containing STGG media (skim milk, tryptone, glucose and glycerol) until processing.

### Samples processing

Anovaginal specimens were streaked onto chromogenic media CHROMagar™ MRSA (CHROMagar, Paris, France) and incubated for 24 h at 37 °C. After this period, one representative colony of each morphotype was selected, based on colony size and color, and identified using MALDI-TOF MS (Matrix-Assisted Laser Desorption Ionization—Time of Flight Mass Spectrometry; Bruker Microflex LT, Bruker Daltonics, Bremen, Germany). Following identification, strains were stored in skim-milk with glycerol (10%) at − 20 °C.

### MRS strains analysis

All strains were confirmed for methicillin resistance phenotype using the disk diffusion method with cefoxitin disc (30 µg) and oxacillin disc (1 µg), according to CLSI^40^. Antimicrobial susceptibility was assessed in all strains also in accordance with CLSI recommendations^40^. Five antimicrobial agents were selected for this testing: erythromycin (15 µg), clindamycin (2 µg), tetracycline (30 µg), sulfamethoxazole/trimethoprim (23,75/1,25 µg) and levofloxacin (5 µg). The D-zone test was used to determine constitutive MLS_B_ (cMLS_B_), inducible MLS_B_ (iMLS_B_), lincosamide (L) or macrolide (M) resistance phenotype, according to CLSI^40^. Multidrug resistance phenotype was defined as resistance to at least three different classes of antibiotics.

Methicillin-resistant strains had their DNA extracted for *mecA* gene detection and SCC*mec* typing, using QIAamp DNA Mini Kit (QIAGEN, Hilden, Germany), according to the manufacturer’s instructions.

Detection of the *mecA* gene was accessed in accordance with Del Vecchio et al.^41^. For determining the SCC*mec* types, multiplex PCR reactions were performed as presented by Kondo et al.^42^.

### Statistical analysis

Statistical analyses were performed using GraphPad Prism version 8.0.2 (GraphPad Software, Boston, MA, USA). Chi-square and Fisher’s test were applied and p-value less than 0.05 was considered significant.

### Institutional review board

The study was conducted in accordance with the Declaration of Helsinki and approved by the local Ethics Committee of University Hospital Clementino Fraga Filho of the Federal University of Rio de Janeiro (UFRJ) (protocol code 43389321.9.0000.5257 and date of approval January 2020).

### Informed consent

Informed consent was obtained from all subjects involved in the study.

## Data Availability

The data presented in this study are available in the present article “Increase in Methicillin-Resistant *Staphylococcus* spp. Colonization among Pregnant Individuals During COVID -19 Pandemic” or available on request from the corresponding author.
